# A hybrid approach to study large conformational transitions of biomolecules from single particle XFEL diffraction data

**DOI:** 10.3389/fmolb.2022.913860

**Published:** 2022-08-29

**Authors:** Han Asi, Bhaskar Dasgupta, Tetsuro Nagai, Osamu Miyashita, Florence Tama

**Affiliations:** ^1^ Department of Physics, Nagoya University, Nagoya, Japan; ^2^ Division of Biological Data Science, Research Center for Advanced Science and Technology, The University of Tokyo, Meguro City, Japan; ^3^ Department of Advanced Materials Science, Graduate School of Frontier Sciences, The University of Tokyo, Kashiwa, Japan; ^4^ RIKEN Center for Computational Science, Kobe, Japan; ^5^ Institute of Transformative Bio-Molecules, Nagoya University, Nagoya, Japan

**Keywords:** hybrid method, 3D structure modeling, X-ray free-electron laser diffraction data analysis, Gaussian mixture model, Monte-Carlo sampling

## Abstract

X-ray free-electron laser (XFEL) is the latest generation of the X-ray source that could become an invaluable technique in structural biology. XFEL has ultrashort pulse duration, extreme peak brilliance, and high spatial coherence, which could enable the observation of the biological molecules in near nature state at room temperature without crystallization. However, for biological systems, due to their low diffraction power and complexity of sample delivery, experiments and data analysis are not straightforward, making it extremely challenging to reconstruct three-dimensional (3D) structures from single particle XFEL data. Given the current limitations to the amount and resolution of the data from such XFEL experiments, we propose a new hybrid approach for characterizing biomolecular conformational transitions by using a single 2D low-resolution XFEL diffraction pattern in combination with another known conformation. In our method, we represent the molecular structure with a coarse-grained model, the Gaussian mixture model, to describe large conformational transitions from low-resolution XFEL data. We obtain plausible 3D structural models that are consistent with the XFEL diffraction pattern by deforming an initial structural model to maximize the similarity between the target pattern and the simulated diffraction patterns from the candidate models. We tested the proposed algorithm on two biomolecules of different sizes with different complexities of conformational transitions, adenylate kinase, and elongation factor 2, using synthetic XFEL data. The results show that, with the proposed algorithm, we can successfully describe the conformational transitions by flexibly fitting the coarse-grained model of one conformation to become consistent with an XFEL diffraction pattern simulated from another conformation. In addition, we showed that the incident beam orientation has some effect on the accuracy of the 3D structure modeling and discussed the reasons for the inaccuracies for certain orientations. The proposed method could serve as an alternative approach for retrieving information on 3D conformational transitions from the XFEL diffraction patterns to interpret experimental data. Since the molecules are represented by Gaussian kernels and no atomic structure is needed in principle, such a method could also be used as a tool to seek initial models for 3D reconstruction algorithms.

## 1 Introduction

Structural information of the biological molecules is necessary for understanding their functions, and thus determination of their structure is one of the primary interests in biology. To determine the 3D structure of biomolecules, multiple experimental techniques have been developed. X-ray crystallography is the most widely used technique to determine the 3D structure at the atomic level (Shi, 2014; Brooks-Bartlett and Garman, 2015; Kemp and Alcock, 2017). However, since it requires the crystallization of biological molecules, it can be difficult to determine the structure of a wide range of biological molecules that are hard to crystallize, such as insoluble molecules. The emergence of single particle cryo-electron microscopy (cryo-EM) enables the imaging of molecular-sized objects and visualization of different functional states without crystallization (Saibil, 2000). Cryo-EM single-particle analysis has yielded protein structures with increasing levels of detail in recent years (Lyumkis, 2019), even enabling the visualization of individual atoms in a protein to be determined (Nakane et al., 2020; Yip et al., 2020). However, the resolution of cryo-EM is generally still lower than X-ray crystallography (Shoemaker and Ando, 2018).

X-ray free-electron laser (XFEL) is the latest generation of the X-ray source that could become an invaluable technique in structural biology. XFEL can create significantly strong, coherent X-rays in a femto-second pulse form (Emma et al., 2010; Ishikawa et al., 2012; Altarelli and Mancuso, 2014), enabling “diffraction before destruction” (Neutze et al., 2000; Gaffney and Chapman, 2007) strategy. With XFEL, it is becoming possible to observe the inner structure of the biological molecules in near-physiological states at room temperature without crystallization or cryo-cooling (Seibert et al., 2011; Kimura et al., 2014; Takayama et al., 2015; van der Schot et al., 2015; Miyashita and Joti, 2017; Spence, 2017). Theoretically, it has been shown that high-resolution 3D structures can be obtained using millions of single particle diffraction patterns (Loh and Elser, 2009; Tegze and Bortel, 2012; Tokuhisa et al., 2012). However, the high-resolution 3D reconstruction is extremely challenging so far because of the poor scattering power of the biological macromolecules and the limited amount of experimental data. Currently, only a small number of low-resolution structures from single-particle approaches have been reported (Gallagher-Jones et al., 2014; Xu et al., 2014; Ekeberg et al., 2015; Hosseinizadeh et al., 2017; Lundholm et al., 2018; Rose et al., 2018; Kobayashi et al., 2021).

Given the current limitations to the resolution and the amount of data available from the single-particle XFEL scattering experiments, it is difficult to reconstruct 3D models *ab initio*. In such a situation, *hybrid approaches*, which combine the computational simulations with experimental data, could be used to obtain information on 3D structures (Alber et al., 2008; van den Bedem and Fraser, 2015; Miyashita and Tama, 2018; Rout and Sali, 2019; Srivastava et al., 2020). In these approaches, computational methods are used to generate hypothetical models that most likely represent the experimental data. Examples of such applications include the recovery of structural details from small-angle X-ray scattering (SAXS) profiles (Gorba et al., 2008; Ravikumar et al., 2013; Kikhney and Svergun, 2015; Kikhney et al., 2016; Schindler et al., 2016; Ekimoto and Ikeguchi, 2018) and cryo-EM data (Tama et al., 2004; Jolley et al., 2008; Topf et al., 2008; Trabuco et al., 2008; Grubisic et al., 2010; Vashisth et al., 2012; Wu et al., 2013; Jin et al., 2014; McGreevy et al., 2016; Miyashita et al., 2017). Such approaches for XFEL data have also been proposed (Tokuhisa et al., 2016; Wang and Liu, 2017), which showed that XFEL diffraction patterns could be used to assess the plausibility of hypothetical conformations.

In this paper, we propose a new hybrid approach to study biomolecular conformational transitions by combining a single 2D low-resolution XFEL diffraction pattern with a known conformational model. Our method derives conformational changes of biomolecules by flexibly deforming an initial low-resolution 3D model to match a target XFEL diffraction pattern using Monte-Carlo (MC) sampling. We refine the initial model iteratively during the MC sampling to maximize the similarity between the XFEL diffraction patterns simulated from candidate models and the target XFEL diffraction pattern. The basic assumption is that the similarity between diffraction patterns correlates reasonably well with the similarity of 3D models, while it is expected that multiple 3D models could match equally well to the target diffraction pattern. Such a strategy can avoid the difficulties of 3D reconstruction from limited data sets along with phase retrieval procedures. Dasgupta et al. developed an algorithm following the same strategy for atomic force microscopy (AFM) studies (Dasgupta et al., 2020, 2021) to recover structural details of the conformational transitions from AFM experimental data. However, for adaptation to XFEL data, many changes were needed, such as calculation and similarity detection of simulated XFEL diffraction patterns.

The proposed algorithm was tested on two biomolecular complexes of different sizes with different complexities of conformational transitions, adenylate kinase, and elongation factor 2 using synthetic XFEL diffraction patterns. Our results show that the proposed algorithm can successfully describe the conformational transitions by refining the overall 3D shapes of biomolecules against a single simulated XFEL diffraction pattern. In addition, we showed that the incident beam orientation has some effect on the accuracy of the 3D structure modeling and discussed why the accuracy is low in some orientations. The proposed method could serve as an alternative approach for retrieving 3D information from the XFEL diffraction, which provides new insight into the interpretation of experimental data. Since the molecules are represented by Gaussian kernels and no atomic structure is needed in principle, this method could also be used as a tool to seek initial models for 3D reconstruction algorithms.

## 2 Materials and methods

### 2.1 Model systems

**FIGURE 1:**
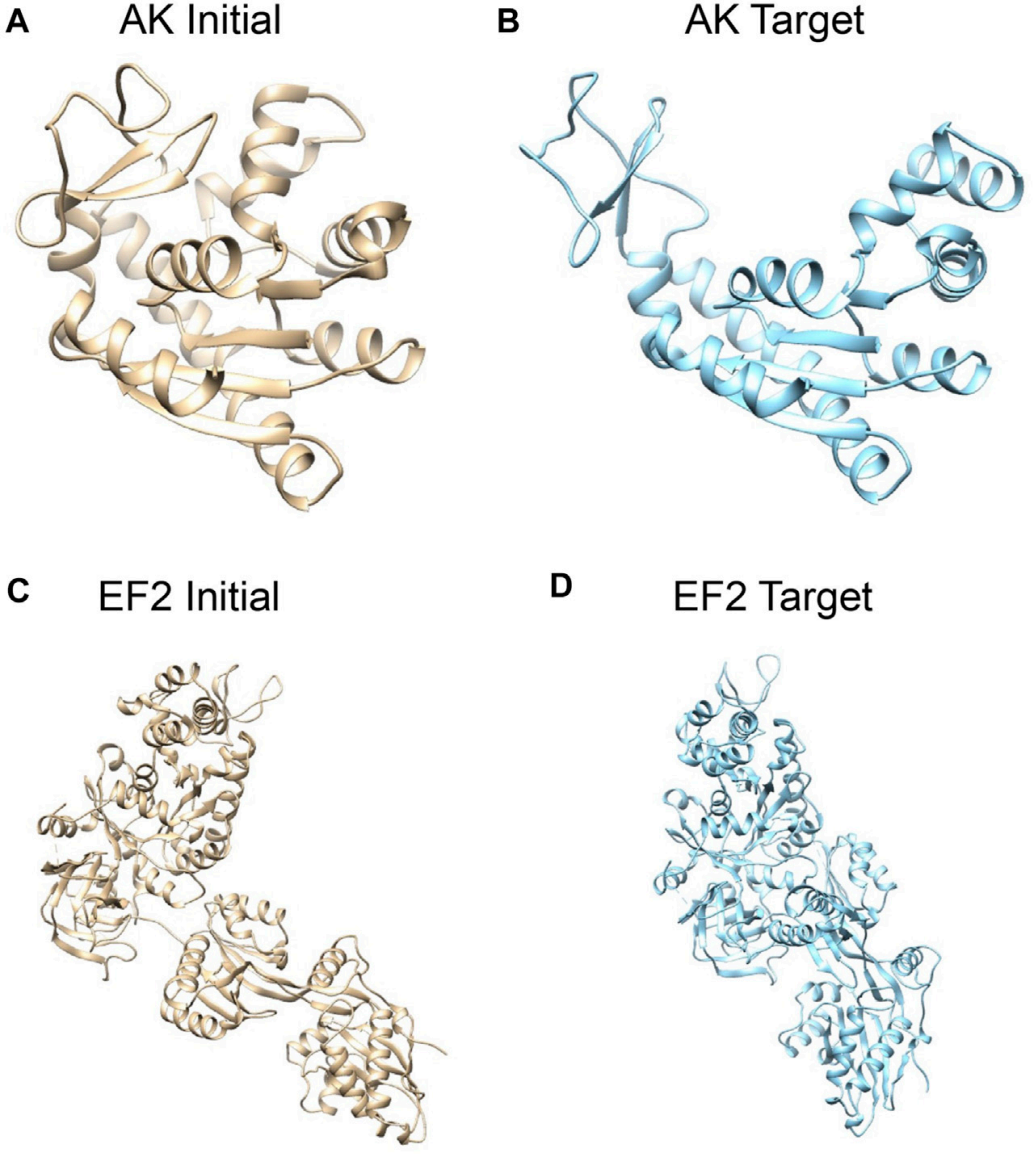
Systems studied: structures of adenylate kinase (AK); **(A)** the initial conformation (PDB: 1ake, chain A) (Whitford et al., 2007) and **(B)** the target conformation (4ake, chain A) (Müller et al., 1996). Structures of elongation factor 2 (EF2); **(C)** the initial conformation (1n0u, chain A) (Jørgensen et al., 2003) and **(D)** the target conformation (1n0v, chain C) (Jørgensen et al., 2003). The initial conformations are superposed to target conformations by Chimera (Pettersen et al., 2004).

To evaluate our proposed algorithm, we performed tests on two proteins (Figure 1). For each protein, two conformations were considered, one arbitrarily assigned to the initial conformation and the other one to the target conformation. The model optimization process is driven by a target XFEL diffraction pattern and uses a MC scheme to change the conformation to match the target XFEL diffraction pattern. The test target XFEL diffraction patterns were synthetic XFEL diffraction patterns generated from target conformations, which were assumed unknown in the tests. However, for evaluating the modeling results, the 3D conformations of the target structures were used to compare against the modeled 3D conformation.

First, we consider *E. coli* adenylate kinase (AK), a protein comprising 214 residues. Two X-ray structures with different conformations, PDB 1ake (Whitford et al., 2007) (closed state) and 4ake (Müller et al., 1996) (open state) differing by 7Å RMSD (backbone atoms), were assigned to the initial conformation and target conformation, respectively (Figures 1A,B).

The second system is elongation factor 2 (EF2), a protein that consists of 842 residues. Two X-ray structures of EF2, PDB 1n0u (Jørgensen et al., 2003) and 1n0v (Jørgensen et al., 2003), differing by 14.4Å RMSD, were assigned to the initial conformation and target conformation, respectively (Figures 1C,D).

### 2.2 Coarse-grained atomic model by Gaussian kernels

In the MC conformational sampling process, we need to efficiently generate a large number of simulated diffraction patterns from structure models in order to compare the 3D candidate models to a target XFEL diffraction pattern. In addition, the current target systems in XFEL experiments are large macromolecular complexes, where atomic details are not essential, and our aim is to provide a method to describe large-scale protein conformational transitions. Therefore, we employ Gaussian mixture models (GMMs) as a coarse-grained approach to represent the structures instead of atomically detailed models. GMMs were found able to capture shape details at a specified resolution (Kawabata, 2008). Furthermore, in the proposed approach, the GMMs were considered as electron densities, which diffract the coherent X-ray beam. We have shown that this approach enables fast calculations of simulated diffraction patterns (Section 2.3 for details) (Nagai et al., 2018).

In the Gaussian mixture model (GMM), a macromolecule is represented by the sum of $N_{g}$ Gaussian distributions. A molecule is represented as density function

$$\begin{array}{c} f\left(r|\Theta \right)=\overset{N_{g}}{\underset{i=1}{\sum }}\pi_{i}\phi \left(r|\mu_{i},\Sigma_{i}\right) \end{array}$$ (1)

where r denotes a position in three-dimensional real space, $\phi \left(r|\mu_{i},\Sigma_{i}\right)$ is the *i*th Gaussian distribution in three-dimensional space, $\pi_{i}$ is its weight, and Θ indicates the set of parameters for describing $N_{g}$ Gaussians. The sum of the weights $\pi_{i}$ is set to be 1:

$$\overset{N_{g}}{\underset{i=1}{\sum }}\pi_{i}=1.$$

The individual Gaussian distributions are written as

$$\begin{array}{c} \phi \left(r|\mu_{i},\Sigma_{i}\right)=\;{\left(2\pi \right)}^{-3/2}{\left|\Sigma_{i}\right|}^{-1/2}\exp\left[-2^{-1}{\left(r-\mu_{i}\right)}^{T}\Sigma_{i}^{-1}\left(r-\mu_{i}\right)\right] \end{array}$$ (2)

where $\mu_{i}$ is the mean position and $\Sigma_{i}$ is the covariance matrix of the distribution, and $\left|\Sigma_{i}\right|$ is the determinant of the matrix $\Sigma_{i}$ .

**FIGURE 2:**
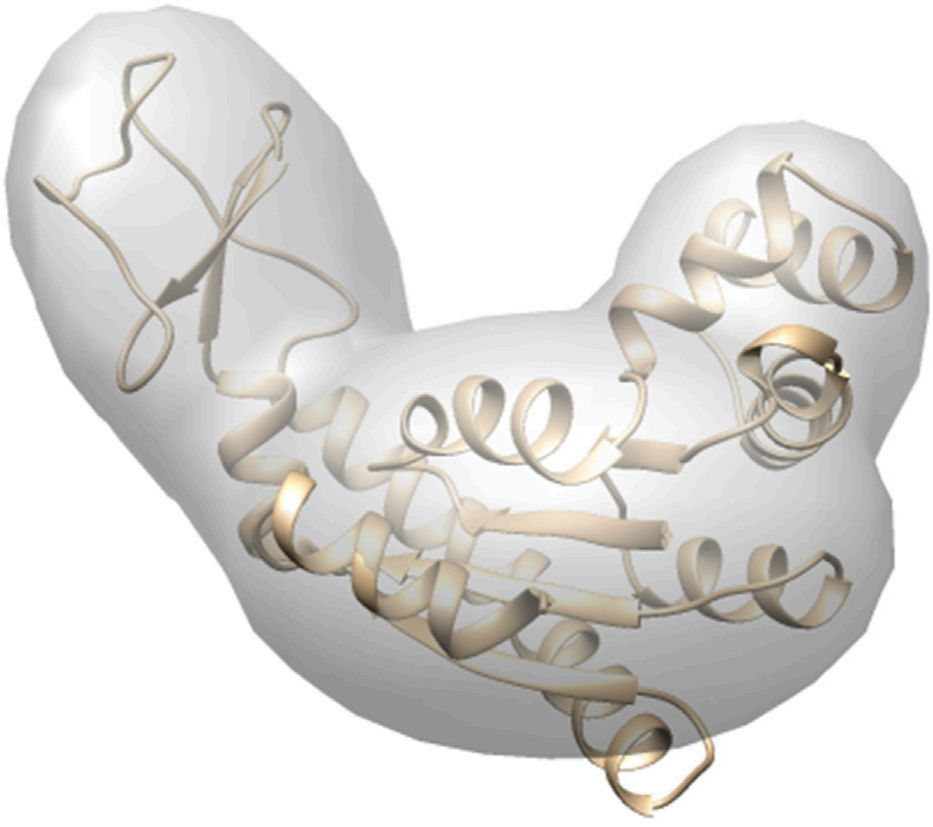
Coarse-grained model by GMM. The number of Gaussian kernels is 3, made from AK target conformation. The domains are defined according to Table 1. The 3 individual kernels are shown with the embedded atomic structure. The kernels were rendered by UCSF Chimera (Pettersen et al., 2004).

One advantage of the proposed approach is that Gaussian kernels can be flexibly defined to describe molecular structures at different scales. We used *gmconvert* (Kawabata, 2008) to obtain the optimized GMM for generating the target diffraction pattern. For a given macromolecule, *gmconvert* uses an expectation-maximization method to estimate the parameter Θ . We used residue-level-kernel GMMs, where *N*_*g*_ is set to be the number of residues, for simulating target diffraction patterns in the tests to simulate the electron density of the protein molecule as accurately as possible. However, for structure modeling, to decrease the dimensionality of the problem to avoid overfitting and higher computational costs, we consider the domain level kernels instead of residue level kernels. In addition, in GMM optimization by *gmconvert* (Kawabata, 2008), there is no guarantee that the kernels are defined in relation to the structural domains conserved during conformational transitions. Therefore, we define the Gaussian kernels of the initial model aligned with protein domains (domain level kernels) to capture the conformational dynamics better. The set of residues included in each structural domain is given in Table 1. For each of these domains, we defined one kernel. The kernel center is defined as the center of the domain, while the covariance matrix is obtained from the atomic coordinates of the domain. The weight of a kernel is defined to be the mass-fraction of the included atoms (Figure 2). The reference GMMs of target conformations for evaluation for modeling results were also generated following the same protocol.

**TABLE 1:** Tested proteins and definition of residue blocks.

Proteins	Number of residues in the initial conformation	Residues to define structural domains
Adenylate kinase	214	1–29, 68–117, 161–214
		30–67
		118–160
Elongation factor 2	819	3–48, 74–221, 329–344
		67–73, 345–485
		222–270
		271–328
		486–561
		562–569, 722–828
		570–721, 829–842

### 2.3 Calculation of diffraction patterns

The Fourier transformation of GMM can be performed analytically, which enables rapid computation of diffraction patterns; the structure factor of GMM is given by

$$\begin{array}{c} F\left(s\right)=\int \int \int f\left(r|\Theta \right)e^{is\cdot r}dr\\ =\overset{N}{\underset{i=1}{\sum }}\pi_{i}e^{is\cdot \mu_{i}}\exp\left[\frac{1}{2}s^{T}\Sigma_{i}s\right], \end{array}$$ (3)

where s represents a diffraction wave vector. Hereafter, we use k=s/(2π) (Nagai et al., 2018).

In our formalism, we assumed that the incident beam comes from the positive side of Z-axis, i.e., the wave vector of the incident beam is (0, 0, $-k_{inc}$ ), that the object is at (0, 0, *d*), and that the detector is set perpendicular to Z-axis with its center being at (0, 0, 0), where *d* is the distance between object and detector (*d* > 0). In elastic scattering, the intensity of diffraction at (*x, y,* 0), *I* (*x, y*)*,* is proportional to ${\left|F\left(k_{x},k_{y},k_{z}\right)\right|}^{2}$ such that

$$\begin{array}{c} k_{x}=k_{inc}\frac{x}{\sqrt{d^{2}+x^{2}+y^{2}}}, \end{array}$$ (4)

$$\begin{array}{c} \begin{array}{c} k_{y}=k_{inc}\frac{y}{\sqrt{d^{2}+x^{2}+y^{2}}}, \end{array} \end{array}$$ (5)

$$\begin{array}{c} k_{z}=k_{inc}-\sqrt{k_{\text{i}nc}^{2}-k_{x}^{2}-k_{y}^{2}}. \end{array}$$ (6)

The coordinates ( $k_{x},k_{y},k_{z}$ ) form half of the Ewald sphere. We obtained two-dimensional diffraction pattern *I* (*x, y*) relative to the intensity at the image center, and, in the following, the diffraction patterns are discussed using *k* values corresponding to pixels as the coordinates, i.e., $I\left(x,y\right)\to I\left(k_{x},k_{y}\right)$ .

**FIGURE 3:**
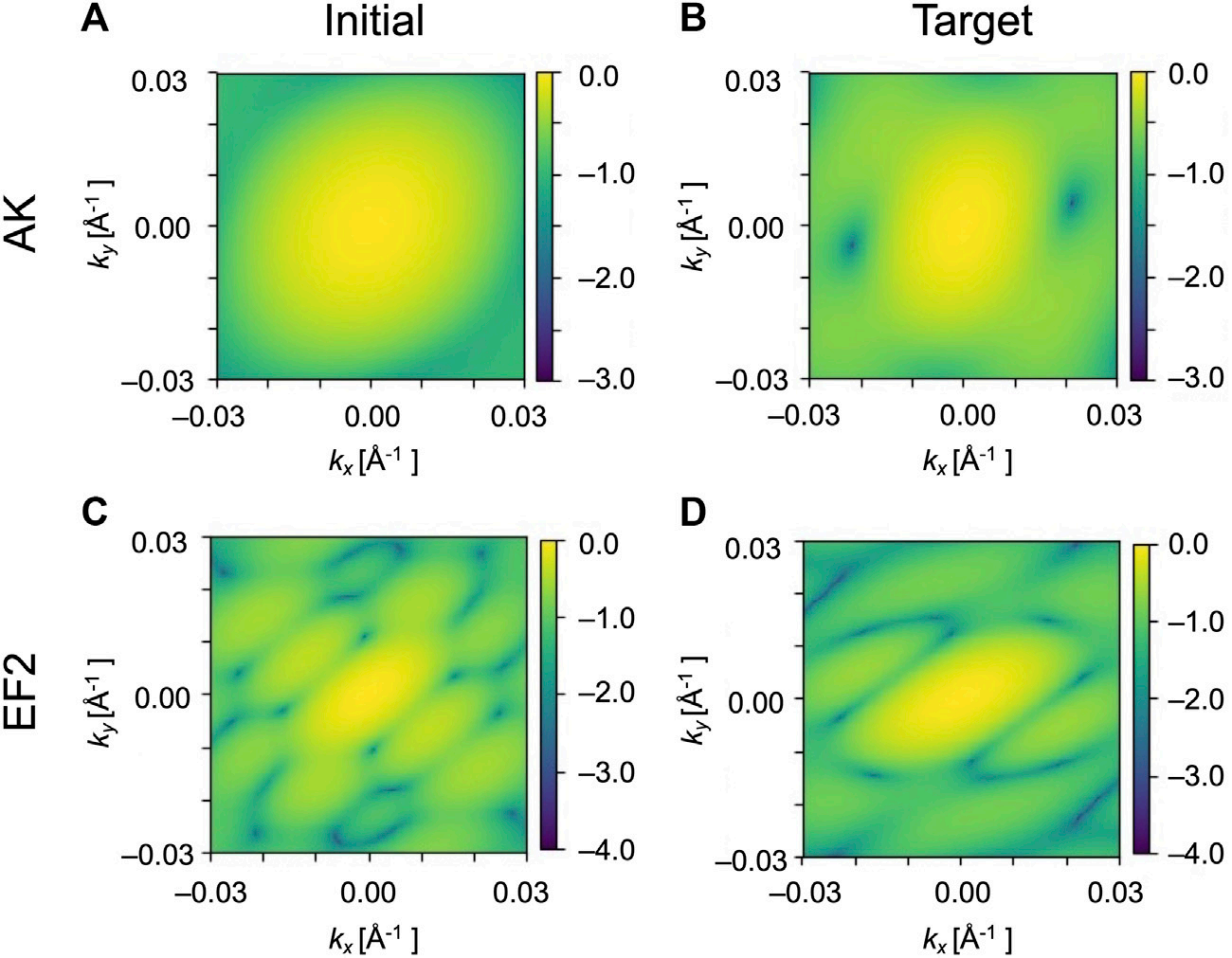
Simulated XFEL diffraction patterns of GMM models from structures shown in Figure 1. The patterns of the initial structure of AK **(A)** and of the target structure **(B)**. The patterns of EF2 for the initial **(C)** and the target **(D)**. *k*_*x*_ and *k*_*y*_ represent the wavenumbers of diffraction vectors (without 2π). The intensities in the diffraction images are shown as log_10_ [*I*(***k***)/*I* (**0**)].

In all the diffraction calculations, the wavelength of the incident beam was set to 1 Å, i.e., $k_{inc}$ = 1 ${\text{Å}}^{-1}$ . The resolution of the diffraction pattern is (0.0003 Å^–1^/pixel)^2^ near the center. Following the above protocol, we simulated the initial and target diffraction patterns for AK and EF2 (Figure 3) by converting atomic structures shown to GMM models as in Figure 1. Noise is not considered in this theoretical work, as we focus on describing large conformational transitions of biomolecules. We use a low-resolution coarse-grained model, GMM, to describe molecular structures, and accordingly, we use only the part of diffraction patterns close to the center where the signal is strong and the effect of noise is weak.

### 2.4 Correlation coefficient between diffraction patterns

In our modeling process, a critical step for accurate modeling is the computation of the correlation coefficient between diffraction patterns simulated from candidate GMM models and the target diffraction pattern. We used Pearson’s correlation coefficient to compare the diffraction patterns. To enhance the sensitivity of the similarity detection during MC sampling, we consider the circle matching region instead of comparing the whole pattern (Tokuhisa et al., 2016; Nagai et al., 2018).

We first obtained the logarithm of the intensity of two diffraction patterns as a function of polar coordinates *k* and ϕ , i.e., $\log_{10}I_{1}\left(k,\phi \right)$ and $\log_{10}I_{2}\left(k,\phi \right)$ . Then we calculated the Pearson’s correlation coefficient between $\log_{10}I_{1}\left(k,\phi \right)$ and $\log_{10}I_{2}\left(k,\phi \right)$ for a resolution ring *k* and defined it as *CC*_*k*_. Each *k* value defines a circle on the diffraction pattern and we used *N* number of *k* values spacing by Δk , i.e., *N* equidistant circles on the diffraction pattern with a circle spacing of Δk . Finally, the diffraction patterns simulated from candidate and target GMM models are compared using the similarity score that we defined as

$$CC_{2D}=\left(1/N\right)\overset{N}{\underset{i=1}{\sum }}CC_{i}.$$ (7)

**FIGURE 4:**
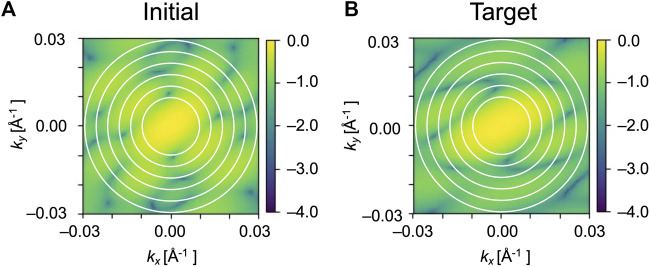
Circle matching region in the diffraction patterns. Only the circles filled with white were used for the calculations of Pearson correlation. **(A)** and **(B)** are simulated diffraction patterns of initial and target GMM models of EF2, respectively. The radii of the circles on the patterns are 0.010, 0.014, 0.018, 0.022, 0.026 and 0.030 Å^−1^.

We used *N* = 6 and *∆k* = 0.004 Å^-1^ in this study (Figure 4).

### 2.5 Monte-Carlo optimization

In our modeling process, we optimize the initial model based on the target XFEL diffraction pattern simulated from the target conformation (Figures 3B,D). This optimization is performed by Monte-Carlo (MC) update of the Gaussian kernel parameters using the Metropolis scheme. In this scheme, the positions and orientations of the Gaussian kernels are randomly updated (from state *j* to *k*), and then corresponding XFEL diffraction patterns are simulated (*j*th and *k*th patterns). Then the updated *k*th representation (or candidate) is accepted or rejected based on the correlation coefficient between *k*th pattern to the target pattern and *j*th to the target pattern. The Metropolis scheme depends on the difference between correlation coefficients at an arbitrary temperature *T*. In this study, *T* was set to 1.0.

In the above scheme, Gaussian kernels should not extensively overlap each other during conformation transitions. Therefore, repulsive interactions between the kernels during the updates are also considered. The overlap between two three dimensional Gaussian kernels $\phi_{i}\left(r|\mu_{i},\Sigma_{i}\right)$ and $\phi_{j}\left(r|\mu_{j},\Sigma_{j}\right)$ is given by

$$\begin{array}{c} \xi_{ij}=\frac{1}{{\left(2\pi \right)}^{3/2}\cdot {\left|\Sigma_{ij}\right|}^{1/2}}\exp\left[-\frac{1}{2}{\left(\mu_{i}-\mu_{j}\right)}^{T}\Sigma_{ij}^{-1}\left(\mu_{i}-\mu_{j}\right)\right], \end{array}$$ (8)

where $\Sigma_{ij}=\Sigma_{i}+\Sigma_{j}$ , $\left|\Sigma_{ij}\right|$ is the determinant of the matrix $\Sigma_{ij}$ (Kawabata, 2008). This overlap is normalized to (Dasgupta et al., 2020)

$$\begin{array}{c} \xi_{ij}^{N}=\xi_{ij}/\sqrt{\xi_{ii}\xi_{jj}}. \end{array}$$ (9)

Before performing the Metropolis method with two-dimensional comparison, candidate models in which any of the normalized overlaps between the pairs of kernels was greater than a threshold $\xi_{max}$ were rejected. The threshold parameter $\xi_{max}$ is decided based on the maximum overlap correlation value from the initial GMM models.

### 2.6 Evaluation of optimized 3D models

Our algorithm uses the MC scheme to refine the initial model based on the assumption that increases in diffraction patterns similarity correlate reasonably well with increases in the 3D model similarity between the candidate and target representations. Therefore, to evaluate the performance of the algorithm, we measured the 3D similarity between the initial model and the target structure (initial 3D CC) and the 3D similarity between the final candidate models and the target structure (final 3D CC). The 3D similarities were calculated using the ‘Fit in Map’ tool in Chimera (Pettersen et al., 2004) to superimpose the maps. We only applied the translation when fitting the maps. The correlation is given by

$$CC_{3D}=\frac{\langle u,v\rangle }{\left|u\right|\left|v\right|},$$ (10)

where u and v are the vectors containing the fit map values and corresponding reference map values.

## 3 Results and discussion

### 3.1 Modeling adenylate kinase open conformation from closed conformation

**FIGURE 5:**
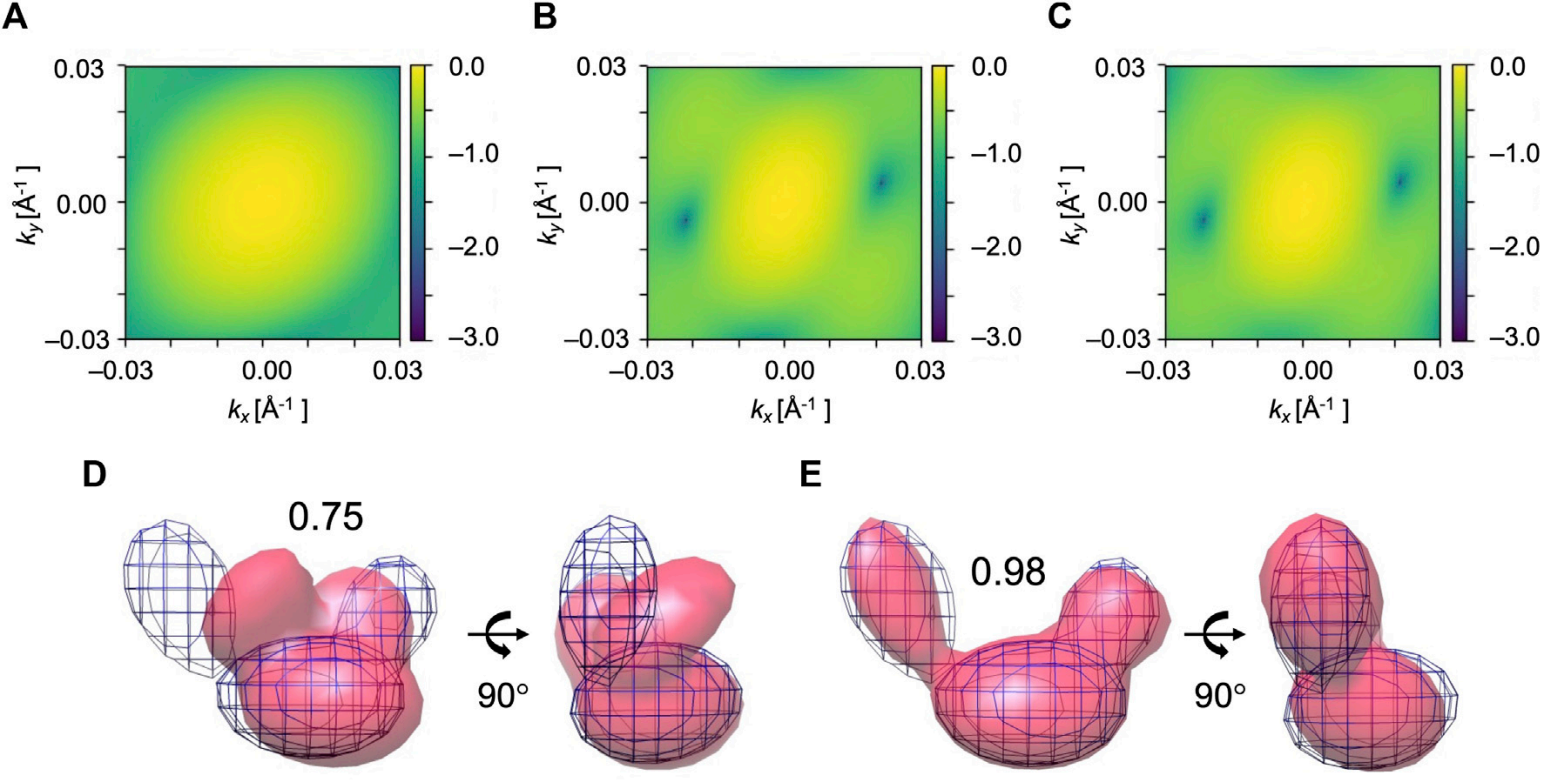
Simulated XFEL diffraction patterns of initial **(A)**, candidate **(B)**, and target **(C)** GMM models from the fitting of AK. *k*_*x*_ and *k*_*y*_ represent the wavenumbers of diffraction vectors (without 2π). Initial 2D CC between **(A)** and **(C)** was 0.17, which increased to 0.99 between the **(B)** and **(C)** after the MC simulation process. GMM representations of AK before and after the fitting are shown. **(D)** The initial model is in pink, the reference GMM representation of the target model is in blue mesh. Initial 3D CC between the initial model and target is 0.75. Incident beam orientation is directed into the plane of paper for the left-side structure and from right to left for the right-side structure. **(E)** The candidate model is in pink, the reference GMM representation of the target model is in blue mesh. The final 3D CC between the candidate model and target is 0.98.

The algorithm was tested on a large conformational transition observed between two conformations of AK. Our aim is to obtain a low-resolution model of the open state AK conformation (PDB ID: 4ake) from the corresponding XFEL diffraction pattern. The initial model is the closed state AK conformation (PDB ID: 1ake). Since AK has three distinct domains, we used 3 Gaussian kernels to represent the initial model (see domain definition in Table 1 and Figure 1). The GMM models at the start of the optimization are shown in Figure 5D.

We performed 10 fitting trials using the simulation parameters given in Table 2. 2D CC between the diffraction patterns increased significantly from 0.17 to 0.99 for all the trajectories (Table 3). Regarding the 3D models, 3D CC increased from 0.75 to the average value of 0.91 for 10 trajectories (Table 3). This result proves our assumption that the diffraction pattern’s similarity correlates reasonably well with the 3D model’s similarity. The highest final 3D CC among the 10 trajectories is 0.98 (Table 3). Such a high final 3D CC value indicates that it is possible to reconstruct 3D volumes of AK open conformation with sufficient accuracy. The resulting best candidate model is shown in Figure 5.

**TABLE 2:** Simulation parameters.

Simulated XFEL diffraction pattern	Excluded volume restraint	Kernel random update	Monte-Carlo scheme
Circle boundary: 1.1–0.03 Å^-1^	Overlap threshold ( ξ ): 0.100 (AK)	Maximum translation: 1.0 Å	Temperature: 1.0
			Steps: 100,000 (AK)
Circle number: 6	0.175 (EF2)	Maximum rotation: 10.0°	
Circle spacing: 0.004 Å^-1^			50,000 (EF2)

**TABLE 3:** Summary of the AK modeling results.

System studied	Number of Gaussians	Number of trials	2D CC			3D CC		
			Initial	Average	Maximum	Initial	Average	Maximum
AK	3	10	0.17	0.99 ± 0.00	0.99	0.75	0.91 ± 0.04	0.98

### 3.2 Modeling elongation factor 2 apo conformation from holo conformation

**FIGURE 6:**
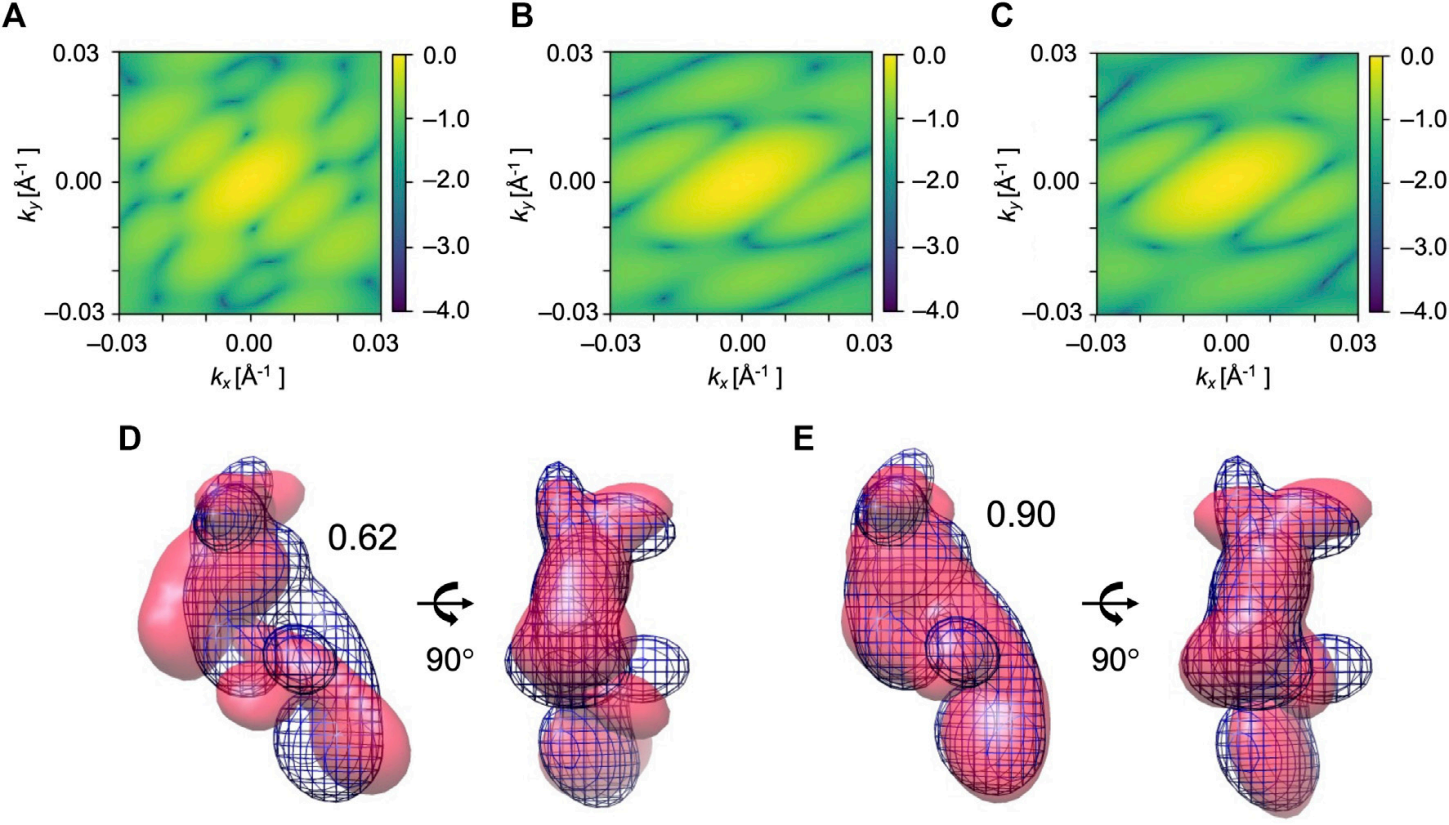
Simulated XFEL diffraction patterns from the initial **(A)**, candidate **(B)**, and target **(C)** GMM models from the fitting of EF2. *k*_*x*_ and *k*_*y*_ represent the wavenumbers of diffraction vectors (without 2π). Initial 2D CC between **(A)** and **(C)** was 0.09, which increased to 0.97 between the **(B)** and **(C)** after the MC simulation process. GMM representations of EF2 before and after the fitting are shown. **(D)** The initial model is in pink, the reference GMM representation of the target model is in blue mesh. Initial 3D CC between the initial model and target is 0.62. **(E)** The candidate model is in pink, the reference GMM representation of the target model is in blue mesh. The final 3D CC between the candidate model and target is 0.90.

The transition observed between two conformations of EF2 is a hinge-type motion with an RMSD of 14.4 Å. The initial model is the holo conformation (PDB ID: 1n0v), and the target is apo conformation (PDB ID: 1n0u). The reference diffraction pattern was simulated from the GMM model of the target conformation with 819 Gaussian kernels, which corresponds to one kernel per residue. Low-resolution models with 7 Gaussian kernels built from the holo and apo conformations (see domain definition in Table 1 and Figure 1) were used as the initial and the reference target model, respectively (Figure 6D).

We performed 10 trial runs using the simulation parameters given in Table 2. 2D CC between the diffraction patterns increased significantly from 0.09 to the average value of 0.94 for 10 trajectories (Table 4). Accordingly, 3D CC increased from 0.62 to the average value of 0.81 for 10 trajectories (Table 4). 3D CC between the best candidate model and the target is 0.90 (Table 4). The resulting best candidate model is shown in Figure 6. Combined with the result of AK, we conclude that the Monte-Carlo strategy has worked for XFEL, and our approach could successfully fit an initial low-resolution 3D model to a target XFEL diffraction pattern.

**TABLE 4:** Summary of the EF2 modeling results.

System studied	Number of Gaussians	Number of trials	2D CC			3D CC		
			Initial	Average	Maximum	Initial	Average	Maximum
EF2	7	10	0.09	0.94 ± 0.04	0.97	0.62	0.81 ± 0.05	0.90

### 3.3 Modeling from X-ray free-electron laser diffraction patterns of adenylate kinase in different views

An XFEL diffraction pattern represents a particular view of the biomolecule, and generally, the orientation of the molecule cannot be controlled. Therefore, it is important to investigate how critical the orientation of the observed molecule can be for the modeling results.

**FIGURE 7:**
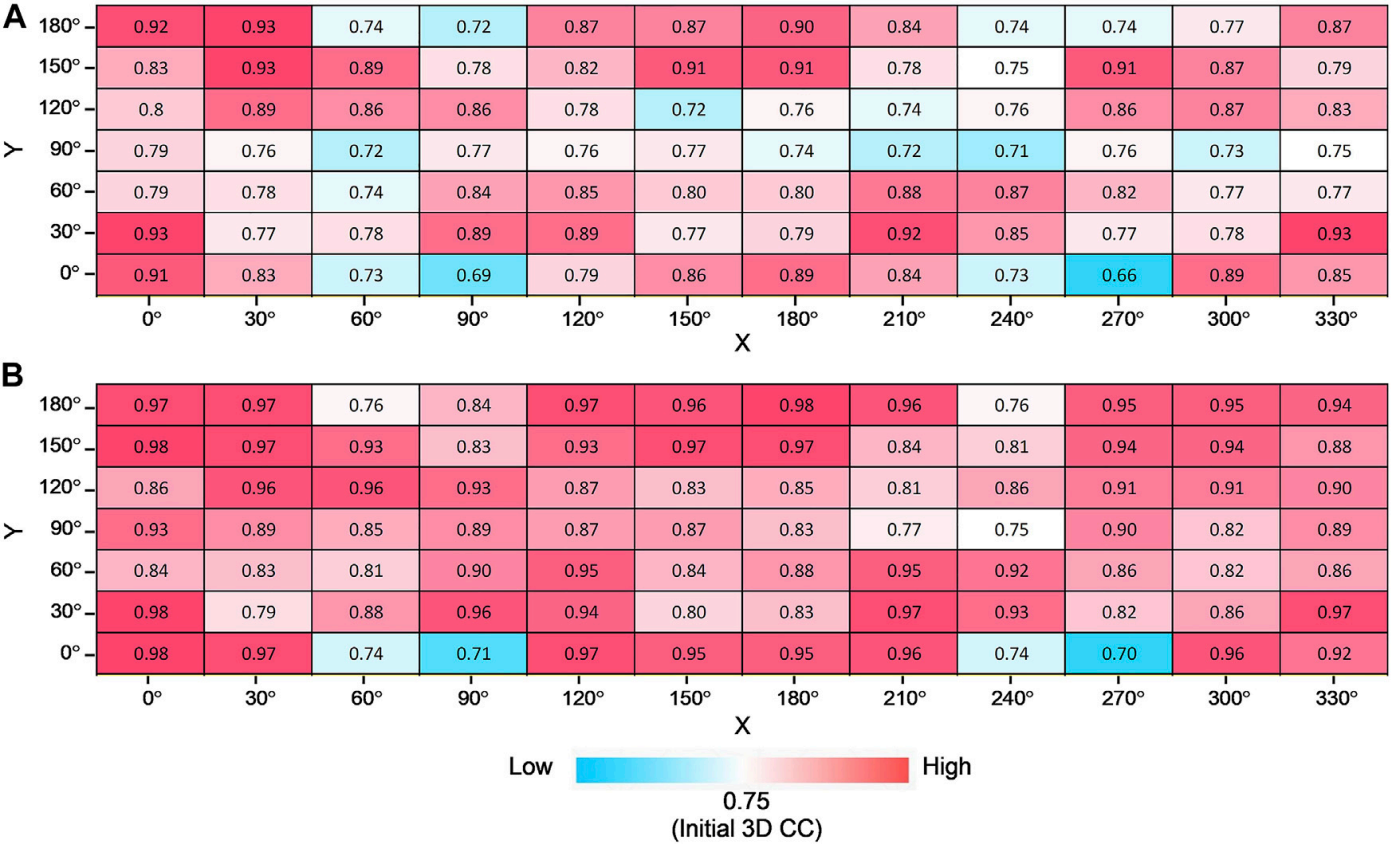
Modeling AK from different orientations. Average **(A)** and the highest **(B)** final 3D CC for 10 trajectories for all combinations of rotation degrees by X-axis and Y-axis with the color scale.

To address this question, we tested the fitting trials for various simulated XFEL diffraction patterns from AK in different orientations. The initial closed state conformation and target open state conformation were rotated by X-and Y-axis (rotation by X-axis is from 0° to 330° in the step of 30°, rotation by Y-axis is from 0° to 180° in the step of 30°) to generate corresponding initial and target GMM representations. In combination, there are 84 modeling cases, and for each orientation, 10 trial runs were performed. The average and highest 3D CC (final) for 84 orientations are shown in Figure 7. We observe that the orientation of the biomolecule has a clear impact on modeling results (Figure 7A). The candidate model with the highest final 3D CC is observed for the orientation X = 0° and Y = 0° (Figure 7B), with 2D CC increased from 0.17 to 0.99 and 3D CC increased from 0.75 to 0.98 (Figure 5). On the other hand, 3D CC did not increase for some orientations. In 52% of the 84 modeling orientations, the highest final 3D CC values are higher than 0.90 (Figure 7B).

### 3.4 Modeling from X-ray free-electron laser diffraction patterns of elongation factor 2 in different views

**FIGURE 8:**
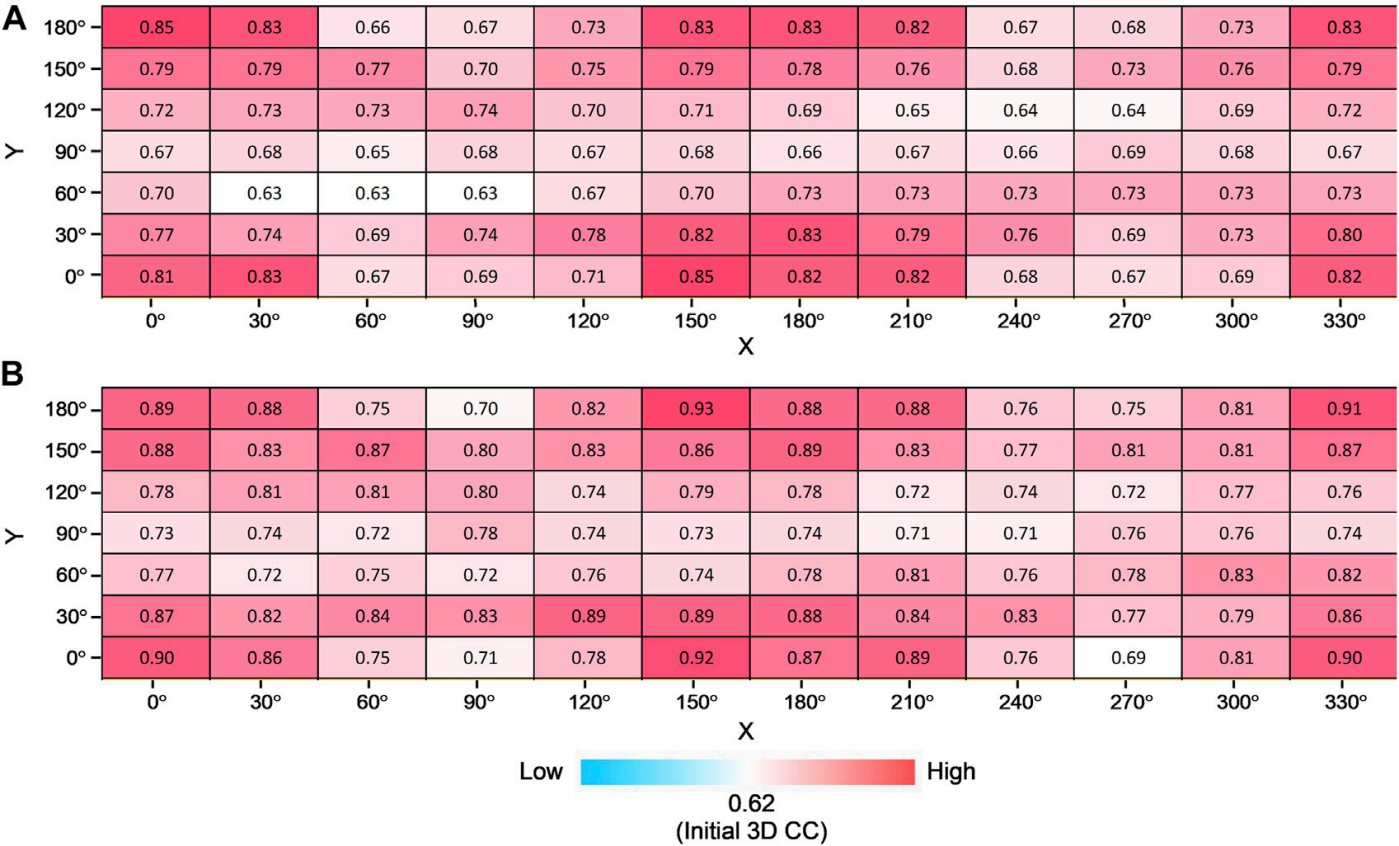
Modeling EF2 from different orientations. Average **(A)** and the highest **(B)** final 3D CC for ten trajectories for all combinations of rotation degrees by X-axis and Y-axis with the color scale.

**FIGURE 9:**
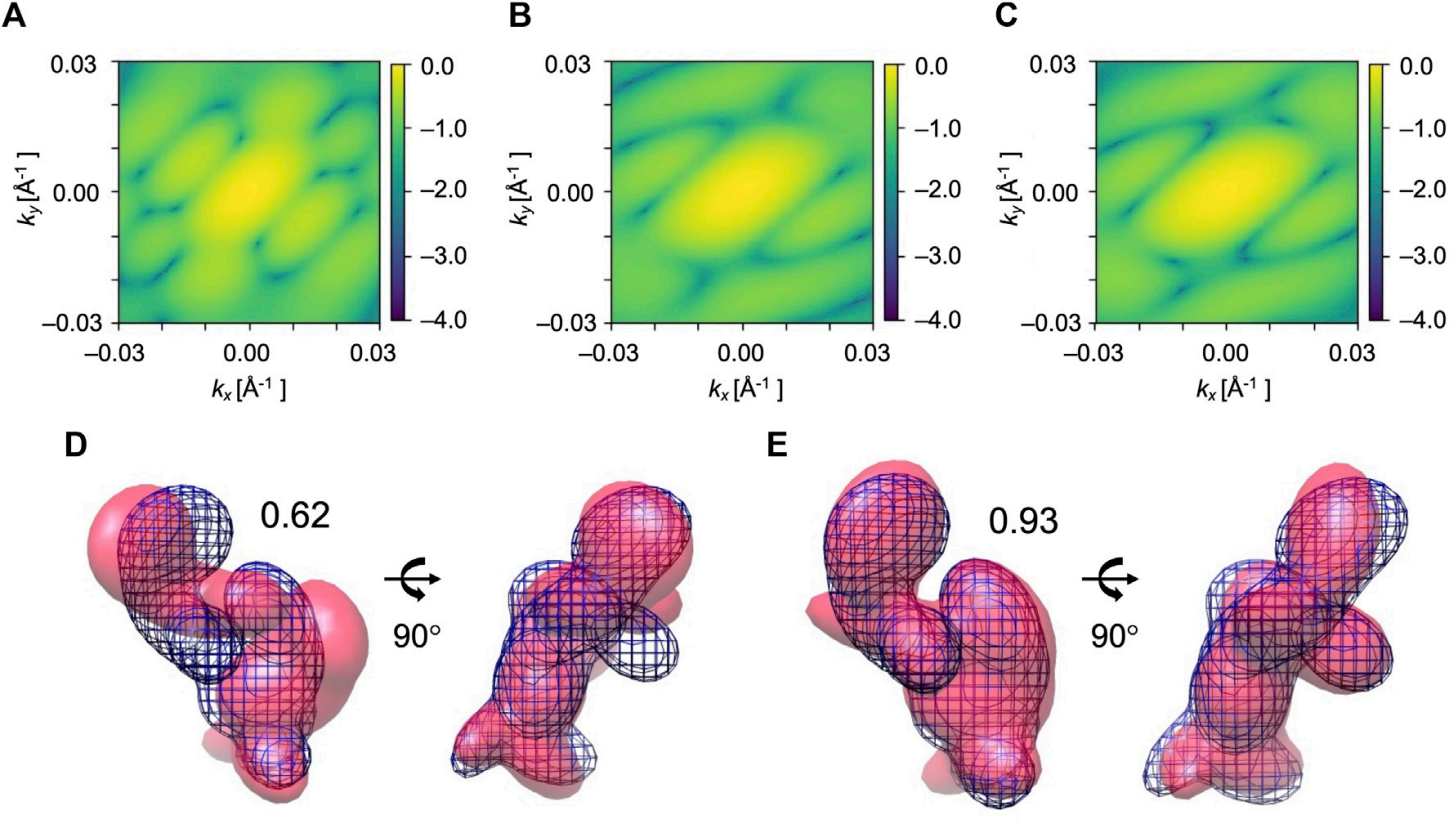
Simulated diffraction patterns from the initial **(A)**, candidate **(B)**, and target **(C)** GMM models from EF2 fitting for X = 150° and Y = 180°. *k*_*x*_ and *k*_*y*_ represent the wavenumbers of diffraction vectors (without 2π). Initial 2D CC between **(A)** and **(C)** was 0.05, which increased to 0.98 between the **(B)** and **(C)** after the MC simulation process. GMM representations of EF2 before and after the fitting for X = 150° and Y = 180° are shown. **(D)** The initial model is in pink, the reference GMM representation of the target model is in blue mesh. Initial 3D CC between the initial model and target is 0.62. **(E)** The candidate model is in pink, the reference GMM representation of the target model is in blue mesh. The final 3D CC between the candidate model and the target is 0.93.

Furthermore, we consider simulated XFEL diffraction patterns from EF2 in different orientations. The initial holo conformation and target apo conformation were rotated by the same rule as the AK test. The average and highest 3D CC (final) for 84 orientations are shown in Figure 8. Similar to the AK test, we can observe that the orientation of the biomolecule has a clear impact on modeling results (Figure 8A). The highest 3D CC between the candidate and the target models is observed for the orientation corresponding to X = 150° and Y = 180° (Figure 8B), with 2D CC increased from 0.05 to 0.98, and 3D CC increased significantly from 0.62 to 0.93 (Figure 9).

**FIGURE 10:**
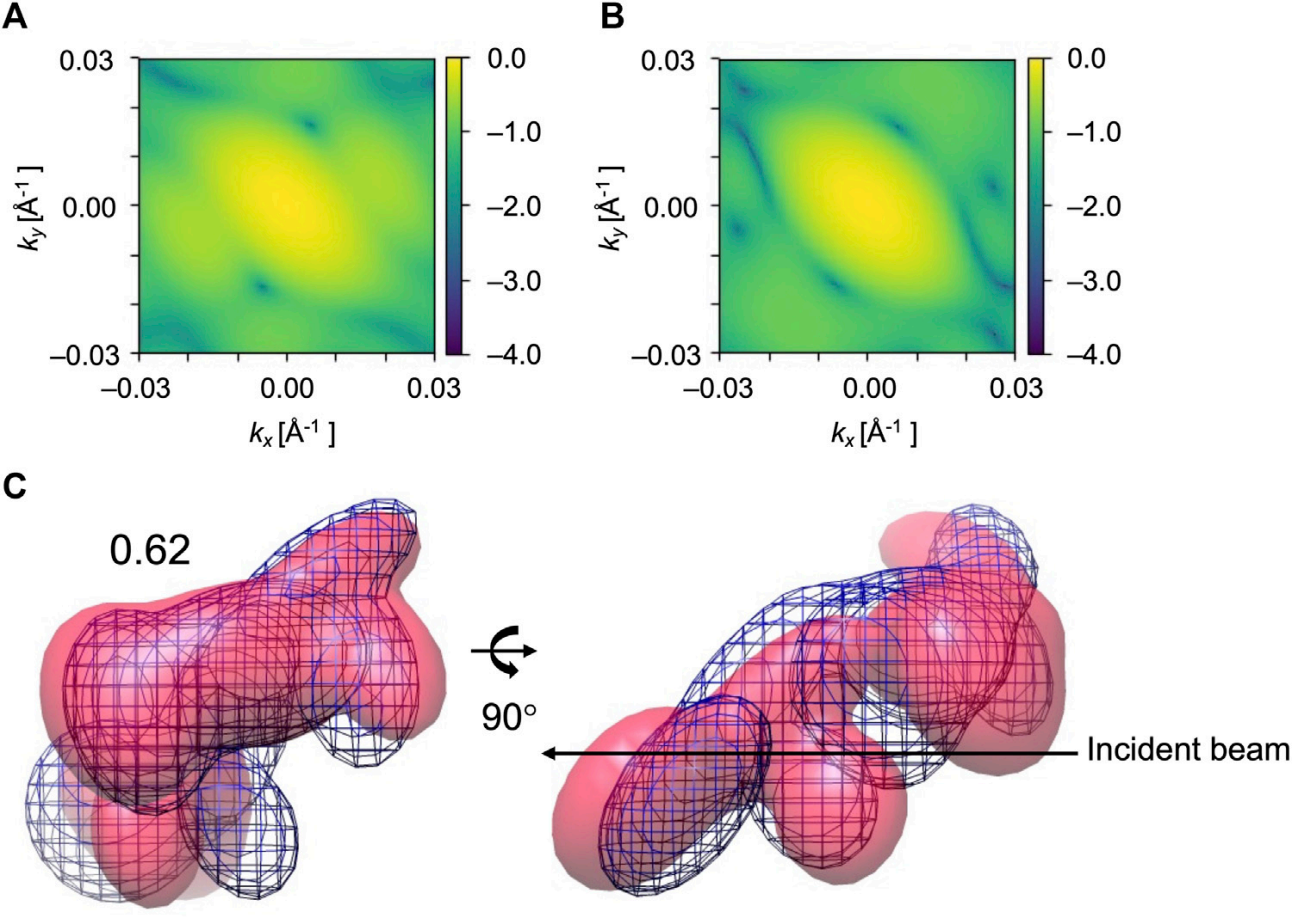
Diffraction patterns and GMM representations of initial and target conformations of EF2 test obtained for X = 60° and Y = 60°, one of the orientations that did not work well. **(A)** and **(B)** are simulated XFEL diffraction patterns from initial and target GMM models, respectively. 2D CC between **(A)** and **(B)** is 0.30. **(C)** The initial model is in pink, the reference GMM representation of the target model is blue mesh. Initial 3D CC between the initial model and target is 0.62.

We could observe from Figure 7A and Figure 8A that 3D CC even decreased in some orientations. This can be explained from the shape of the molecule and the incident beam orientation. For example, X = 60° and Y = 60° is one of the orientations that does not work well for EF2 tests (Figure 8A). The average final 3D CC of the ten trajectories is 0.63, which barely increased from 0.62, the initial 3D CC. We could observe from the GMM representations of the initial and final conformations (Figure 10C) that the middle part has greater densities in the projected orientation, and the diffraction patterns are not sensitive to the repositioning of the kernels along the incident beam path. Thus, conformation details are not well captured in the diffraction pattern for this beam direction. Accordingly, fitting is also strongly affected by the part that has greater densities. Note that a comparably high initial 2D CC (Figures 10A,B) is also observed for this orientation. In contrast, there were no such ‘thick’ parts in orientations that have a clear increase in 3D similarity (Figure 6 and Figure 9) with much more conformational details exposed in the incident beam direction. Accordingly, these orientations have lower initial 2D CC (Figure 6 and Figure 9).

### 3.5 Sensitivities of initial model alignment accuracy

In the proposed structure optimization approach, the initial model needs to have an orientation similar to the orientation that the XFEL diffraction pattern represents. This is a common requirement for flexible fitting approaches, including the fittings to 3D information such as electron density maps. The orientation of a protein molecule observed in XFEL diffraction patterns can also be estimated by calculating correlation coefficients (Tokuhisa et al., 2016; Tiwari et al., 2021). However, such an alignment cannot be exact since the conformations are different and the resolution of the data is low. Therefore, we examined the effect of orientation errors (misalignment) of the initial conformation in relation to the target conformation on the modeling accuracy.

**FIGURE 11:**
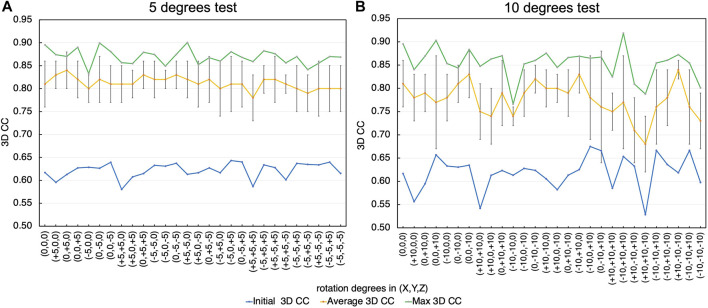
Effect of orientation errors (misalignment) of the initial conformation in relation to the target conformation on the modeling accuracy. **(A)** Rotation by −5, 0, and 5 degrees. The initial 3D CC for each orientation (blue), the average (yellow) and the standard deviations (error bar), and the highest (green) of the final 3D CC values are shown. **(B)** Rotation by −10, 0 and 10 degrees.

For the same target diffraction pattern of EF2 as Section 3.2, we changed the orientation of the initial model by combining rotations around X, Y, and Z-axis by –5, 0, and 5 degrees, and repeated the same test with 10 trial runs (Figure 11A). We also tested the combination of –10, 0, and 10 degrees (Figure 11B). The misalignment by 5 degrees has small effects on the final fitted models. The resulting 3D CC values were between 0.83 and 0.9 (best CC) and 0.78 and 0.84 (average), which are similar to the values obtained when the initial model was pre-aligned using the original atomic models, 0.9 (best CC) and 0.81 (average). When the initial model was misaligned by 10 degrees, some effects on the final CC scores appeared. Although the resulting 3D CC values are comparable to the original alignment for many initial orientations, they are less consistent; the resulting 3D CC values are lower for some initial orientations, especially when the initial structures are misaligned by larger angles. Therefore, applications of this approach to experimental data would require repeated fitting trials starting from the initial orientations with slight variations, and examinations of the obtained models to assess their reliabilities. Nonetheless, the final 3D CC values increased from the initial value for all the cases, demonstrating sufficient robustness of the proposed fitting algorithm.

## 4 Conclusion

In this study, we proposed a new hybrid method that can be applied to single particle XFEL diffraction patterns to produce a low-resolution 3D model and study conformational transitions. In the current single particle XFEL experiments on biological systems, it is still difficult to perform 3D reconstruction directly from the diffraction patterns. We explored the strategies to obtain plausible 3D structural models by optimizing a known structure to maximize the similarity between the target XFEL diffraction pattern and simulated diffraction pattern from candidate models using MC sampling. A set of Gaussian kernels are used to represent the candidate models. There were significant increases in the similarity between the candidate models and target conformation in most cases. Thus, our method could successfully refine initial models of AK and EF2 to infer target conformations using just one XFEL diffraction pattern. In addition, as the biomolecules are represented by Gaussian kernels, this approach could be used to study the conformational changes of the molecules where only low-resolution structural information without atomic details is available. Therefore, the proposed algorithm can be a new approach to studying the dynamics of biomolecules from XFEL experiments, as an addition to the structure determination *via* 3D reconstruction from a large dataset.

## Data Availability

The codes of the method developed in this article can be found at https://github.com/TamaLab/xfel-fitting-gmm.
